# Synthesis and antitumor activities of 3-substituted-analine derivatives: structure modifications of Tuv part of tubulysins

**DOI:** 10.1186/s13065-018-0483-5

**Published:** 2018-11-15

**Authors:** Mingsha Shao, Xinfa Bai, Xuan Ma, Ning Yan, Lei Yao

**Affiliations:** School of Pharmacy, Key Laboratory of Molecular Pharmacology and Drug Evaluation (Yantai University), Ministry of Education, Collaborative Innovation Center of Advanced Drug Delivery System and Biotech Drugs in Universities of Shandong, Yantai University, Yantai, 264005 People’s Republic of China

**Keywords:** Tubulysin, Analogues, Synthesis, Antitumor activity

## Abstract

**Background:**

Tubulysins family is a kind of natural compound with potent, antitumor activity. To simplify the synthesis route and find new antitumor compounds is becoming a hotspot of research recent years.

**Results:**

Starting from 3-nitrobenzoic acid, after 7 steps transformations, 12 new tubulysin analogues were synthesized by the conformational restraint and bioisostere principle. These structures are featuring 3-substituted analine moieties. All these compounds are new compounds, and the structures were characterized by ^1^H NMR, ^13^C NMR, and HRMS. The antitumor activities were screened by the MTT method using MDA-MB-231and MCF7 cells.

**Conclusions:**

Compound **IIb** exhibited certain antitumor activity with the IC_50_ value of 7.6 and 11.8 µM against MDA-MB-231 and MCF7 cells respectively. Compounds **IIa**–**IIe** had moderate antitumor activities suggested that the thiazole ring in the Tuv could be replaced by the phenyl ring. However, Compounds **Ia**–**Ie** lose antitumor activity dramatically suggested that the conformation of the Tuv was crucial for the tubulysin analogues to maintain the biological activity.

**Electronic supplementary material:**

The online version of this article (10.1186/s13065-018-0483-5) contains supplementary material, which is available to authorized users.

## Background

**Fig. 1:**
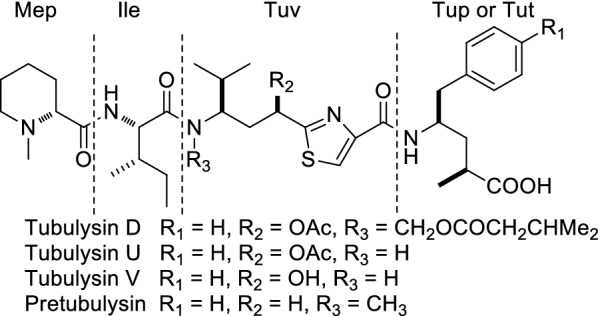
Structures of tubulysins and pretubulysin

Tubulysins (Fig. 1) are natural tetrapeptides isolated by Höfle in 2001 [1]. They possess potent antitumor activities by binding to tubulin near the vinca alkaloid binding site and inhibiting tubulin polymerization [1]. The average IC_50_ against mammalian cancer cells ranged from 0.01 to 10 nM [2]. Due to the high potency and limited availability, there have been several total synthesis routes reported ever since the first total synthesis of tubulysin D by Ellman’s group [3–5]. In 2009, Pretubulysin (Fig. 1), the precursor to tubulysin, was found by Müller from *Angiococcus disciformis* [6]. Although it was less potent than the tubulysin, the anticancer activities still retained subnanomolar level [7–9]. The extraordinary anticancer activity of tubulysin and pretubulysin against a validated target makes them exciting leads for the development of novel drugs for multidrug-resistant cancers.

## Results and discussion

### Chemistry

**Scheme1:**
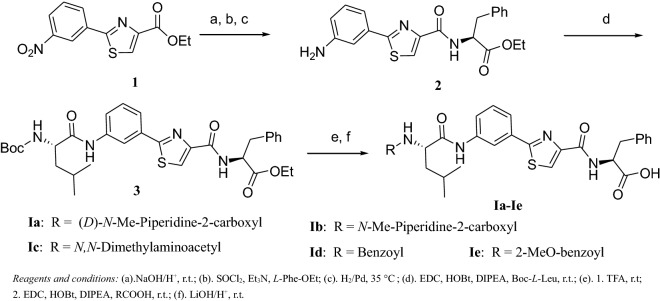
Synthesis of tubulysins derivatives **Ia**–**Ie**

Some issues, such as less lipophilicity, large molecular weight ( > 700) and too many stereocenters, have hampered tubulysin from becoming a commercial drug. In our lab, efforts were taken to modify the structure of tubulysin in order to increase the lipophilicity and decrease the numbers of stereocenters. In all the total synthesis routes of tubulysin, one of the biggest challenges was to build the Tuv part. Utilizing conformational restrain principle, a new series of tubulysin analogues were designed by cyclization and aromatization of the *i*-propyl group and R_2_ group into a phenyl ring (Scheme 1). Some advantages, like decreasing numbers of stereocenters, increasing lipophilicity, and simplifying the synthesis, were obvious to this series of compounds.

Starting from commercially available ethyl 2-aryl-4-thiazole-carboxylate (**1**), after three steps transformation, compound **3** was obtained. The l-phenylalanine was chosen instead of the Tup in the tubulysin, because a previous structure–activity-relationship study showed that the Tup had little effect on the antitumor activity [10, 11]. After two classical peptide coupling reactions and one hydrolysis reaction, compound **Ia**–**Ie** were obtained in moderate yields. To fulfill our design, *l*-leucine and benzoic acids were used in the peptide coupling. Compared with tubulysins, this series of compounds were relatively low molecular weight (around 550), and less stereocenters (Scheme 1).

**Scheme2:**
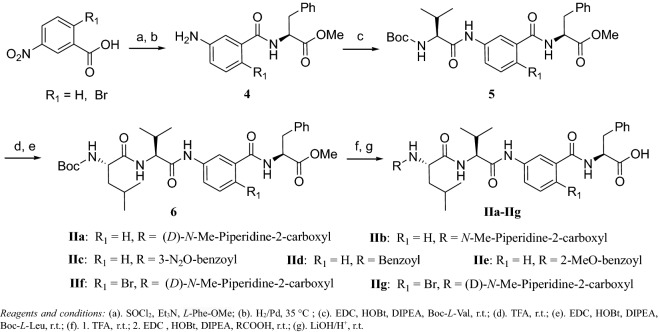
Synthesis of tubulysin derivatives **IIa**–**IIg**

Inspired by the structure of pretubulysin, compounds **IIa**–**IIg** (Scheme 2) were designed and synthesized. In these compounds, peptide bonds were adopted as the linker to connect the Tuv and Tup parts, instead of the ethylene group in pretubulysin. Besides the thiazole group, a phenyl group was also used according to the bioisosterism principle. Another reason that a phenyl group was used was because there were at most 4 positions could be utilized to introduce substituents to modify the pharmacokinetic profiles of the compounds.

The syntheses of compounds **IIa**–**IIg** started from 3-nitrobenzoic acid or 2-bromo-5-nitro-benzoic acid. After the amide bond formation reaction and hydrogenation reaction, compound **4** was obtained and used in the next step to couple with Boc-l-Val directly to obtain compound **5**. Then, by classical peptide coupling conditions, the Leu part and *N*-Mep part were installed subsequently utilizing classical peptide coupling reaction.

### Antitumor activity

Human cancer cell lines Siha, PC3 and MKN45 were cultured in RPMI-1640 media supplemented with 10% fetal calf serum, penicillin (100 U/mL) and streptomycin (100 μg/mL) (Gibco BRL, NY, USA), and incubated at 37 °C in a humidified air atmosphere containing 5% CO_2_. All cells were harvested in their exponentially growing phase.

Cell viability was measured using (MTT) assay. Briefly, Cells were seeded in 96-well multi-plates at a density of 4 × 10^4^/mL. After incubated overnight, triplicate wells were exposed to vehicle and test agents for 72 h. MTT solution (5 mg/mL) was added to each well and incubated continued for 4 h. DMSO was added to dissolve the MTT formazan product and the absorbance was measured at 570 nm using a Molecular Devices SpectraMax M5 (Molecular Devices, USA). The relative cell viability rates were calculated versus untreated controls. The 50% inhibitory concentration (IC_50_) values were calculated using the Graph Pad Prism 5 (Graph Pad Software, Inc., USA). The antitumor activities of all the synthesized compounds were listed in Table 1.

**Table 1:** The antitumor activities of the compounds **Ia**–**IIg**

Compd.	IC_50_ (μm)		Compd.	IC_50_ (μm)	
	MDA-MB-231	MCF7		MDA-MB-231	MCF7
**Ia**	> 20	> 20	**IIa**	17.1	> 20
**Ib**	> 20	> 20	**IIb**	7.6	11.8
**Ic**	> 20	> 20	**IIc**	16.3	> 20
**Id**	> 20	> 20	**IId**	> 20	15.5
**Ie**	> 20	> 20	**IIe**	16.3	> 20
Taxol	< 0.01	0.006	**IIf**	> 20	> 20
			**IIg**	> 20	> 20

The antitumor activities of compound **Ia**–**Ie** were not as expected, while compounds **IIa**–**IIe** had moderate antitumor activities. This suggests that the conformation of the Tuv and Tup parts was crucial to the antitumor activity. In compounds **Ia**–**Ie**, due to the conformational restraint,the Tuv part and Tup part were co-planar and separated far apart, which led to weak or no interaction with tublin. However, in tubulysins and compounds **IIa**–**IIe**, the ethylene or amide bond linker can freely rotate, which results in the Tuv and Tup parts binding better with tublin. This observation was consistent with previously reported results of THP-tubulysins [12, 13]. On the other hand, Tamura [14] reported that cyclotubulysin, an analog with a *N,O*-acetal ring generated from C-11 alcohol and N-14, had a similar antitumor activity with vinblastine. This also suggests that the conformation of Tuv and Tup part was crucial to the antitumor activity. Previous SAR studies showed that the entire Tup residue was not essential for activity [10]. In order to simply the synthesis, *l*-Phe was arbitrarily selected in this study. However, according to Ullrich’s findings [15], compounds **IIa**–**IIe** would increase the activities by replacement of *l*-Phe with Tup. The reason why compound **IIf** and **IIg** had no activities is not clear yet. Although more work is needed to increase the potency, this work shows at the very least that the phenyl ring could be used to replace the thiozole ring of the Tuv.

## Conclusions

Compound **IIb** exhibited certain antitumor activity with the IC_50_ value of 7.6 and 11.8 µM against MDA-MB-231 and MCF7 cells respectively. Compounds **IIa**–**IIe** had moderate antitumor activities suggested that the thiazole ring in the Tuv could be replaced by the phenyl ring. However, Compounds **Ia**–**Ie** lose antitumor activity dramatically suggested that the conformation of the Tuv was crucial for the tubulysin analogues to maintain the biological activity.

## Experimental section

### Chemistry

Reactions employed oven- or flame-dried glassware under nitrogen unless otherwise noted. Thin layer chromatography (TLC) employed glass 0.25 mm silica gel plates with UV indicator. Flash chromatography columns were packed with 230–400 mesh silica gel as a slurry in the initial elution solvent. Gradient flash chromatography was conducted by adsorption of product mixtures on silica gel, packing over a short pad of clean silica gel as a slurry in hexane, and eluting with a continuous gradient as indicated. Nuclear magnetic resonance (NMR) data were obtained at operating frequencies indicated in the text and are reported in units of ppm. Infrared spectra were recorded using a single beam FT-IR spectrophotometer by standard transmission method. Low and high resolution mass spectra (TOF) were obtained from local instrumentation facilities services.

#### (*S*)-Ethyl 2-(2- (3-amiophenyl)thiazole-4-carboxamido)-3-phenylpropanoate (**2**)

Step 1: To a solution of compound **1** (5.56 g, 20 mmol) in 60 mL THF:H_2_O (1:1), was added NaOH aqueous solution (2.40 g, 60 mmol) at room temperature. The reaction mixture was allowed to stir at room temperature for 10 h till TLC showed that starting material all consumed. The solvent was removed by *vaccum*, the residue was adjusted the pH to 2 by 10% HCl. The white precipitate was isolated and washed with water, dried and used in next step with further purification. Step 2: The solid obtained in last step was dissolved in 100 mL DCM, SOCl_2_ (4.5 mL, 60 mmol) and 0.5 mL DMF was added at room temperature. The reaction mixture was allowed to stir at reflux condition for 4 h. The excess SOCl_2_ and DCM were removed under reduced pressure. The residue was diluted with 50 mL DCM and added through an additional funnel to a solution of *l*-Phe-OEt (3.86 g, 20 mmol) , Et_3_N (6.06 g, 60 mmol) in 100 mL DCM at 0 degree. The reaction mixture was allowed to stir at 0 degree for 30 min, then room temperature for 1 h. Water was added the reaction mixture, the aqueous layer was extracted with DCM (100 mL × 3). The organic layers were combined and dried over Na_2_SO_4_. The solvent was filtered, concentrated to afford pale yellow oil, which afforded a white solid after recrystallization from a co-solvent of EA/methanol. Yield: 87%.^1^H NMR (400 MHz, CDCl_3_) δ: 8.79 (t, *J* = 1.9, 1H), 8.34 (ddd, *J* = 8.2, 2.2, 0.9, 1H), 8.29–8.22 (m, 1H), 8.20 (s, 1H), 7.84 (d, *J* = 8.2, 1H), 7.69 (t, *J* = 8.0, 1H), 7.40–7.34 (m, 2H), 7.34–7.30 (m, 1H), 7.27–7.23 (m, 2H), 5.07 (dt, *J* = 8.2, 6.1, 1H), 4.23 (q, *J* = 7.1, 2H), 3.38–3.22 (m, 2H), 1.28 (t, *J* = 7.1, 3H). Step3. The above solid 7.40 g (17.4 mmol) was dissolved in MeOH,then about 0.5 g Pd/C (10%) was added. The reaction mixture was allowed to stir at 35 °C for 7 h under H_2_ (0.3 MPa) condition. After the reaction completed, the catalyst was removed by passing through a pad of *celit*, the filtrate was concentrated, and the residue (6.87 g brown solid) was used in next step directly.

#### (*S*)-Ethyl 2-(2-(3-((*S*)-2-((*tert*-butoxycarbonyl)amino)-4-methylpentanamido)phenyl)thiazole-4-carboxamido)-3-phenylpropanoate (**3**)

To a solution of Boc-l-Leu (4.02 g, 17.4 mmol) dissolved in 40 mL DCM,was added EDC (5.01 g, 26.1 mmol), HOBt (2.35 g, 17.4 mmol), and 1 mL DIPEA at room temperature. The reaction mixture was allowed to stir at room temperature for 1 h,then a solution of compound **4** (6.87 g, 17.4 mmol) in 20 mL DCM was added dropwisely. The reaction mixture stirred for 12 h at room temperature. Water 100 mL was added to the reaction mixture and the aqueous layer was extracted with DCM (100 mL × 3). The organic layers were combined, dried with Na_2_SO_4_, filtered and concentrated. The residue (pale yellow oil 10 g) was purified by chromatography (EA: PE = 1:4) to afford white solid 8.46 g, yield: 80%. ^1^H NMR (400 MHz, CDCl_3_) δ: 9.44 (s, 1H), 8.49 (d, *J* = 7.6, 1H), 7.92 (s, 1H), 7.84 (s, 1H), 7.74 (d, *J* = 7.8, 1H,), 7.36 (d, *J* = 7.3, 1H), 7.29 (s, 2H), 7.26 -7.21 (m, 1H), 7.18 (d, *J* = 7.6, 1H), 5.75 (s, 1H), 5.17 (dd, *J* = 15.0, 7.2, 1H), 4.53 (s, 1H), 4.23 (q, *J* = 7.1, 2H), 3.41–3.22 (m, 2H), 2.02 (d, *J* = 15.8, 1H), 1.82 (ddd, *J* = 25.6, 13.7, 7.0, 3H), 1.46–1.36 (m, 9H), 1.26 (t, *J* = 7.1, 4H), 1.03 (dd, *J* = 13.3, 6.3, 6H).

#### General synthesis method for compound **Ia**–**Ie**

Step 1: To a solution of D-*N*-Methyl-2-pipecolinic acid (0.04 g, 0.26 mmol) in 4 mL DCM,was added EDC (0.08 g, 0.39 mmol), HOBt (0.04 g, 0.26 mmol), 0.5 mL DIPEA at 0 °C. The reaction mixture was stirred for 1 h at room temperature. Meanwhile, a solution of compound **3** (0.16 g, 0.26 mmol) in 4 mL TFA was allowed to stir at room temperature for 3 h. The excess TFA was removed under reduced pressure, the residue was dissolved in 5 mL DCM and0.5 mL DIPEA. This solution was added to above reaction mixture under 20 °C. The reaction mixture was allowed to stir overnight at room temperature. Water 50 mL was added to reaction mixture, the aqueous layer was extracted with DCM (40 mL × 3). The organic layers were combined, dried with Na_2_SO_4_, filtered and concentrated. The residue was purified by chromatography (EA:PE = 1:4) to afford white solid 0.13 g, yield: 81%.

Step 2: To a solution of above solid 0.11 g (0.17 mmol) in THF:H_2_O = 1:1 (10 mL, v:v), was added LiOH (0.02 g, 0.83 mmol). The reaction mixture was allowed to stir at room temperature till TLC showed that all starting material consumed. Solvent was removed under reduced pressure, the residue was diluted with water 50 mL, and adjusted with 10% HCl to pH = 2, extracted with DCM (40 mL × 3). The organic layers were combined, dried with Na_2_SO_4_, filtered and concentrated. The residue was purified by chromatography (Chloroform: MeOH = 7: 1) to afford white solid 80 mg, yield: 77%. ^1^H NMR (CD_3_OD, 400 MHz) δ: 8.19 (s, 1H), 8.07 (s, 1H), 7.61 (dd, *J *= 16.0, 8.0, 2H), 7.35 (t, J = 7.9, 1H), 7.16 (dd, *J* = 17.6, 7.2, 4H), 7.08 (t, *J* = 6.8, 1H), 4.66–4.59 (m, 1H), 4.45 (dd, *J *= 9.8, 4.6, 1H), 3.30–3.25 (m, 1H), 2.97 (m, 1H), 2.69 (s, 3H), 1.83 (m, 3H), 1.66 (m, 6H), 1.51 (d, *J *= 12.5, 2H), 1.33–1.14 (m, 1H), 0.91 (dd, *J* = 10.8, 6.1, 6H); HRMS calcd for C_32_H_39_N_5_O_5_S [M+H]^+^ 606.2750, found 606.2755.

**Ib**: white solid, yield: 80%.^1^H NMR (CD_3_OD, 400 MHz) δ: 8.29 (s, 1H), 8.16 (d, *J *= 2.5, 1H), 7.71 (d, *J *= 6.6, 1H), 7.64 (t, *J* = 7.3, 1H), 7.44 (t, *J *= 7.9, 1H), 7.31–7.22 (m, 4H), 7.19 (d, *J* = 6.5, 1H), 4.63 (m, 1H), 4.45 (dd, *J *= 9.8, 4.6, 1H), 3.30–3.25 (m, 1H), 2.97 (m, 1H), 2.69 (s, 3H), 1.83 (m, 3H), 1.66 (m, 6H), 1.51 (d, *J* = 12.5, 2H), 1.33–1.14 (m, 1H), 0.91 (dd, *J *= 10.8, 6.1, 6H); ^13^C NMR (CD_3_OD, 100 MHz) δ: 20.3, 22.0, 24.7, 29.6, 37.9, 40.5, 42.8, 52.6, 54.3, 55.2, 55.9, 68.4, 115.3, 117.5, 118.2, 121.8, 123.2, 126.1, 127.8, 129.3, 133.2, 137.7, 139.1, 150.2, 161.0, 161.4, 161.7, 167.9, 171.9; HRMS calcd for C_32_H_39_N_5_O_5_S [M+H]^+^ 606.2750, found 606.2755.

**Ic**: white solid, yield: 79%.^1^H NMR (CD_3_OD, 400 MHz) δ: 8.23 (d, *J *= 1.7, 1H), 8.09 (s, 1H), 7.65 (d, *J* = 7.7, 1H), 7.59 (dd, *J* = 8.2, 1.1, 1H), 7.36 (t, *J *= 8.0, 1H), 7.18 (d, *J* = 4.4, 4H), 7.11 (m, 1H), 4.58–4.45 (m, 1H), 3.93 (q, *J* = 15.6, 1H), 3.34–3.23 (m, 1H), 3.12 (m, 1H), 2.84 (s, 6H), 1.62 (m, 3H), 1.24–1.16 (m, 2H), 0.92 (t, *J *= 6.2, 6H);^13^C NMR (CD_3_OD, 100 MHz) δ: 20.4, 22.0, 24.6, 36.9, 40.6, 43.0, 52.9, 57.8, 117.5, 121.9, 122.1, 123.7, 126.4, 128.1, 129.1, 132.9, 136.6, 138.9, 149.7, 161.2, 164.5, 168.1, 171.8; HRMS calcd for C_29_H_35_N_5_O_5_S [M+H]^+^ 566.2437, found 566.2434.

**Id**: white solid, yield: 75%. ^1^H NMR (CD_3_OD, 400 MHz) δ: 8.25 (s, 1H), 8.06 (s, 1H), 7.84–7.86 (m, 2H), 7.58–7.62 (m, 1H), 7.42–7.47 (m, 1H), 7.31–7.35 (m, 4H), 7.16–7.18 (m, 2H), 6.97–7.08 (m, 1H), 6.95–6.97 (m, 2H), 4.74–4.78 (m, 2H), 3.11–3.2 (m, 1H), 1.69–1.74 (m, 3H), 0.94–0.96 (d, *J *= 6.2, 6H), 0.78–0.80 (m, 1H);^13^C NMR (CD_3_OD, 100 MHz) δ: 20.9, 22.2, 36.9, 41.3, 50.9, 53.1, 117.7, 120.7, 121.0, 121.9, 122.0, 123.7, 126.5, 128.1, 129.0, 129.4, 130.8, 133.1, 136.6, 139.0, 139.6, 149.6, 161.7, 166.4, 168.1, 172.2, 174.9; HRMS calcd for C_32_H_32_N_4_O_5_S [M+H]^+^ 585.2093, found 585.2104.

**Ie**: white solid, yield: 81%. ^1^H NMR (CD_3_OD, 400 MHz) δ: 8.36 (s, 1H), 8.17 (d, *J *= 3.3, 1H), 7.96 (dd, *J *= 7.8, 1.7, 1H), 7.70 (dd, *J *= 11.7, 4.6, 2H), 7.58–7.48 (m, 1H), 7.44 (t, *J *= 7.9, 1H), 7.32–7.25 (m, 4H), 7.18 (t, *J* = 7.1, 2H), 7.08 (t, *J* = 7.5, 1H), 4.89–4.81 (m, 2H), 4.01 (s, 3H), 3.38 (d, *J* = 5.1, 1H), 3.25 (dd, *J *= 13.8, 7.6, 1H), 1.91–1.75 (m, 2H), 1.24 (dd, *J *= 13.3, 6.1, 1H), 1.06 (d, *J* = 5.8, 6H);^13^C NMR (CD_3_OD, 100 MHz) δ: 20.9, 22.1, 24.9, 36.9, 41.4, 53.1, 55.3, 111.6, 117.6, 120.6, 121.0, 121.9, 122.1, 123.7, 126.5, 128.1, 129.0, 129.4, 130.8, 133.1, 133.2, 136.6, 139.1, 149.6, 157.8, 166.4, 168.0, 172.1; HRMS calcd for C_33_H_34_N_4_O_6_S [M+H]^+^ 615.2277, found 615.2281.

#### (*S*)-Methyl 2-(3-aminobenzamido)-3-phenylpropanoate (**4**)

Step 1: To a solution of 3 nitrobenzoic acid (16.71 g, 100 mmol) in 200 mL DCM, was added SOCl_2_ (14 mL, 200 mmol) and 0.5 mL DMF at room temperature. The reaction mixture was allowed to stir at reflux condition for 5 h. The excess solvent was removed under reduced pressure, the residue was diluted with DCM 50 mL, and added via an additional funnel to a solution of *l*-Phe-OMe (17.91 g, 100 mmol), Et_3_N (30.6 g, 300 mmol) in DCM 100 mL at 0 °C. The reaction mixture was allowed to stir at 0 °C for 0.5 h, and then room temperature for 1 h till TLC showed that the reaction completed. Water 200 mL was added to the reaction mixture, the aqueous was extracted with DCM (150 mL × 3). The organic layers were combined, dried, filtered, and concentrated. The residue was re-crystallized from a co-solvent of ethyl acetate and methanol (1:6) to afford a white solid 29.19 g (yield 89%) and used in next step directly. Step 2: To a solution of above compound (29.15 g, 88.9 mmol) in 200 mL methanol, was added 1 g Pd/C (10%). The reaction mixture was allowed to stir under H_2_ (0.3 MPa) condition at 35 °C for 7 h. The reaction mixture was filtered through a pad of *Celite*, the filtration was concentrated to afford a gray solid 27.77 g as crude product, which was purified via re-crystallization from methanol and ethyl acetate (1:5) 24.77 g (93%). ^1^H NMR (400 MHz, CDCl_3_) δ 7.30–7.33 (m, 4H), 7.17–7.22 (m, 4H), 7.13–7.15 (m,1H), 6.89 (m, 1H), 6.38 (m, 1H), 5.10–5.12 (m, 1H), 3.80 (s, 3H), 3.31 (dd, *J* = 14.0, 5.8 Hz, 1H), 3.24 (dd, *J* = 14.0, 5.9 Hz, 1H). ^13^C NMR (CDCl_3_, 100 MHz) δ: 37.9, 52.4, 53.5, 113.9, 116.7, 118.3, 127.2, 128.6, 129.3, 135.0, 135.5, 135.8, 146.5, 167.0, 172.0; HRMS calcd for C_17_H_18_N_2_O_3_ [M+H]^+^ 299.1317, found 299.1314.

#### (*S*)-Methyl 2-(5-amino-2-bromobenzamido)-3-phenylpropanoate (**4a**)

Made by the same method as described in compound **4**. ^1^H NMR (400 MHz, CDCl_3_) δ 7.30–7.34 (m, 6H), 7.21–7.26 (m, 1H), 6.68 (s, 1H), 6.61–6.65 (m, 2H), 5.09–5.13 (m, 1H), 3.81 (s, 3H), 3.22–3.36 (m, 2H). ^13^C NMR (CDCl_3_, 100 MHz) δ: 37.8, 51.8, 53.7, 116.0, 117.9, 122.0, 127.2, 128.6, 129.4, 133.8, 134.2, 167.0, 146.6, 166.5, 171.2; HRMS calcd for C_17_H_17_BrN_2_O_3_ [M+H]^+^ 377.0423, found 377.0418.

#### (*S*)-Methyl 2-(3-((*S*)-2-(9 (*tert*-butoxycarbonyl)amino)-3-methylbutanamido)benzamido)-3-phenylpropanoate (**5**)

To a solution of Boc-l-Val (8.68 g, 40.0 mmol) in 60 mL DCM, was added EDC (9.98 g, 52.0 mmol), HOBt (5.40 g, 40.0 mmol), and 3 mL DIPEA at 0 °C. The reaction mixture was allowed to stir room temperature for 1 h. Then a solution of compound **6** (11.92 g, 40.0 mmol) in DCM was added to above reaction mixture. The resulting reaction mixture was stirred at room temperature for 1 h. Water 150 mL was added, and the aqueous layer was extracted with DCM (120 mL × 3). The organic layers were combined, dried, filtered, and concentrated. The residue was purified by a chromatography (EA: PE = 1:4) to afford white solid 16.70 g (yield: 84%). ^1^H NMR (400 MHz, CDCl_3_) δ 9.18 (s, 1H), 7.63–7.71 (m, 2H), 7.18–7.34 (m, 6H), 7.08 (m, 1H), 6.97 (m, 1H), 5.83–5.86 (m, 1H), 5.18–5.19 (m, 1H), 4.30–4.34 (m,1H), 3.78 (s, 3H), 3.26–3.28 (m, 2H), 2.17–2.18 (m, 1H), 1.56 (s, 9H), 1.04–1.14 (m, 6H). ^13^C NMR (100 MHz, CDCl_3_) δ: 19.2, 28.3, 31.0, 38.1, 52.3, 53.7, 60.9, 80.7, 117.3, 122.1, 123.0, 126.9, 128.5, 128.9, 129.3, 134.8, 136.5, 137.3, 167.3, 168.3, 171.4, 172.7; HRMS calcd. for C_27_H_35_N_3_O_6_[M+H]^+^ Exact Mass: 498.2526, found 498.2538.

#### (*S*)-Methyl2-(2-bromo-5-((*S*)-2-(tert-butoxycarbonyl)-3-methylbutanamido)benzamido)-3-phenylpropanoate (**5a**)

Made by the same method as described in compound **5**. ^1^H NMR (CDCl_3_, 400 MHz) δ: 9.17 (br s, 1H), 7.74 (m, 1H), 7.49–7.51 (m, 1H), 7.30–7.32 (m, 5H), 7.12–7.14 (m, 1H), 6.81 (br s, 1H), 6.01 (m, 1H), 5.15–5.21 (m, 1H), 4.28 (m, 1H), 3.81 (s, 3H), 3.25–3.35 (m, 2H), 2.07–2.17 (m, 1H), 1.47 (s, 9H), 1.06–1.11 (m, 6H). ^13^C NMR (CDCl_3_, 100 MHz) δ: 19.1, 28.3, 29.7, 38.2, 52.3, 53.6, 60.7, 82.1, 113.9, 117.4, 118.9, 121.1, 127.0, 128.5, 129.4, 133.8, 136.1, 136.7, 157.3, 165.3, 166.6, 172.3. HRMS calcd for C_27_H_34_BrN_3_O_6_ [M+H]^+^ 576.1631, found 576.1628.

#### (*S*)-methyl 2-(3-((*S*)-2-((S)-2-((*tert*-butoxycarbonyl)amino)-4-methylpentanamido)-3-methylbutanamido)benzamido)-3-phenylpropanoate (**6**)

To a solution of Boc-l-Leu (4.50 g, 19.5 mmol) in 50 mL DCM, was added EDC (4.87 g, 25.4 mmol), HOBt (2.63 g, 19.5 mmol), and 1 mL DIPEA at 0 °C. The reaction mixture was stirred at room temperature for 1 h. Meanwhile, a solution of compound **7** (9.69 g, 19.5 mmol) in 4 mL TFA was stirred for 3 h at room temperature. The access of TFA was removed under reduced pressure. The residue was diluted in 25 mL DCM, and added to above reaction mixture via an additional funnel. The resulting reaction mixture was allowed to stir at room temperature overnight. Water 100 mL was added, and the aqueous layer was extracted with DCM (100 mL × 3). The organic layers were combined, dried, filtered, and concentrated. The residue was purified by a chromatography (EA:PE = 1:3) to afford compound **6** as white solid 9.89 g (yield 83%). ^1^H NMR (400 MHz, CDCl_3_) δ: 9.42 (s, 1H), 8.00 (d, *J *= 7.7, 1H), 7.92 (s, 1H), 7.30 (dd, *J *= 14.6, 7.6, 4H), 7.25 (d, *J* = 7.2, 1H), 7.16 (d, *J* = 6.9, 2H), 6.91 (d, *J *= 7.2, 1H), 6.85 (d, *J *= 7.6, 1 H), 5.74 (s, 1H), 5.06 (dd, *J *= 13.4, 6.0, 1H), 4.67–4.37 (m, 1H), 4.15 (s, 1H), 3.73 (s, 3H), 3.25 (qd, *J *= 13.9, 5.9, 2H), 2.25 (d, *J *= 6.3, 1H), 1.74 (dd, *J *= 13.3, 6.6, 1H), 1.62 (dd, *J* = 9.4, 4.9, 2 H), 1.37 (s, 9H), 1.00 (d, *J* = 6.7, 6H), 0.91 (d, *J *= 6.5, 6H).

^13^C NMR (CDCl_3_, 100 MHz) δ:17.3, 22.2, 22.6, 28.3, 31.1, 37.1, 41.2, 51.9, 52.6, 53.6, 58.6, 79.9, 117.7, 122.1, 123.1, 126.9, 127.9, 128.9, 129.2, 134.9, 138.7, 139.6, 156.1, 167.7, 171.3, 171.6, 172.1. HRMS calcd for C_33_H_46_N_4_O_7_ [M+H]^+^ 611.3366, found 611.3358.

#### (*S*)-Methyl2-(2-bromo-5-((S)-2-((S)-2-(tert-butoxycarbonyl)-4-methylpentanamido)-3-methylbutanamido)benzamido)-3-phenylpropanoate (**6a**)

Made by the same method as described in compound **6**. ^1^H NMR (CDCl_3_, 400 MHz) δ: 9.32 (br s, 1H), 7.89–7.99 (m, 1H), 7.82 (m, 1H), 7.26–7.31 (m, 4H), 7.15–7.17 (m, 2H), 6.84–6.86 (m, 2H), 5.75 (br s, 1H), 4.94–4.98 (m, 1H), 4.56–4.60 (m, 1H), 4.15 (m, 1H), 3.75 (s, 3H), 3.17–3.29 (m, 2H), 2.23–2.25 (m, 1H), 1.74–1.77 (m, 1H), 1.60–1.63 (m, 2H), 1.37 (s, 9H), 0.90–1.01 (d, *J *= 8.0, 12H). ^13^C NMR (CDCl_3_, 100 MHz) δ:17.3, 22.2, 22.5, 28.3, 31.0, 37.1, 41.2, 51.9, 52.7, 53.6, 58.9, 79.7, 118.9, 122.1, 123.0, 126.9, 128.6, 128.9, 129.3, 134.9, 137.7, 139.2, 156.5, 167.7, 171.2, 171.5, 172.3. HRMS calcd for C_33_H_45_BrN_4_O_7_ [M+H]^+^ 689.2472, found 689.2481.

Compounds **IIa**–**IIg** were synthesized by the same method as described in compound **Ia**.

**IIa**: white solid, yield 80%. ^1^H NMR (CD_3_OD, 400 MHz) δ: 7.98 (d, *J* = 8.8, 1H), 7.72 (dd, *J *= 8.0, 7.1, 1H), 7.45 (dd, *J* = 7.7, 1.3, 1H), 7.36 (td, *J *= 7.9, 1.6, 1H), 7.31–7.19 (m, 4 H), 7.15 (td, *J* = 7.1, 1.5, 1H), 4.78–4.63 (m, 1H), 4.53–4.40 (m, 1H), 4.36 (d, *J* = 7.6, 1H), 3.35–3.29 (m, 2H), 2.52 (d, *J* = 2.9, 1H), 2.45 (d, *J* = 2.7, 3H), 2.15 (dd, *J* = 13.9, 6.9, 1H), 1.94 (d, *J* = 8.1, 1H), 1.81 (d, *J* = 13.0, 1H), 1.68 (tdd, *J* = 15.2, 10.9, 4.5, 6H), 0.95 (ddd, *J* = 11.5, 8.5, 4.5, 12H); ^13^C NMR (CD_3_OD, 100 MHz) δ: 17.2, 18.4, 20.3, 20.9, 22.0, 24.6, 29.2, 30.5, 39.9, 42.5, 46.3, 52.0, 54.9, 59.2, 67.8, 118.7, 122.4, 122.6, 125.6, 125.9, 127.7, 128.6, 129.1, 135.3, 138.0, 170.0, 170.6, 171.9, 172.1, 173.2; HRMS calcd for C_34_H_47_N_5_O_6_ [M+H]^+^ 622.3605, found 622.3599.

**IIb**: white solid, yield 79%.^1^H NMR (CD_3_OD, 400 MHz) δ: 7.95 (d, *J *= 12.5, 1H), 7.68 (dd, *J *= 19.6, 8.2, 1H), 7.45 (d, *J* = 7.9, 1H), 7.37 (t, *J *= 7.9, 1H), 7.24 (q, *J *= 7.2, 3H), 7.15 (t, *J* = 6.6, 1H), 4.60–4.47 (m, 1H), 4.48–4.37 (m, 1H), 4.38–4.26 (m, 1H), 3.50 (d, *J* = 9.6, 1H), 3.13 (s, 1H), 2.94–2.72 (m, 1H), 2.15 (ddd, *J* = 40.8, 24.7, 14.9, 2H), 1.86 (dd, *J *= 26.9, 15.5, 2H), 1.69 (dt, *J* = 13.8, 9.6, 4H), 1.10–0.84 (m, 12H). ^13^C NMR (CD_3_OD, 100 MHz) δ: 17.2, 18.4, 20.2, 21.1, 22.0, 24.6, 29.3, 30.9, 40.1, 42.5, 45.9, 52.1, 55.0, 59.2, 67.8, 118.8, 122.4, 122.7, 125.6, 125.9, 127.7, 128.6, 129.3, 135.4, 138.0, 170.1, 170.3, 171.9, 174.4, 174.9. HRMS calcd for C_34_H_47_N_5_O_6_ [M+H]^+^ 622.3605, found 622.3599.

**IIc**: white solid, yield 80%.^1^H NMR (CD_3_OD, 400 MHz) δ: 8.63 (t, *J *= 1.9, 1H), 8.33–8.25 (m, 1 H), 8.15 (d, *J *= 7.8, 1H), 7.63 (d, *J *= 8.0, 1H), 7.61–7.54 (m, 1H), 7.34 (d, *J* = 7.8, 1H), 7.27 (t, *J *= 7.9, 1H), 7.22 –7.13 (m, 4H), 7.12–7.05 (m, 1H), 4.73 (dd, *J* = 9.5, 4.9, 3H), 4.65 (dd, J = 9.4, 4.3, 1H), 4.26 (t, *J* = 6.6, 1H), 3.30 (s, 1H), 3.13–2.93 (m, 1H), 2.07 (dd, *J* = 13.2, 6.7, 1H), 1.64 (ddd, *J* = 26.5, 10.3, 5.4, 3H), 0.99–0.81 (m, 12H); ^13^C NMR (CD_3_OD, 100 MHz) δ: 20.5, 22.0, 24.8, 30.9, 36.8, 40.1, 52.7, 54.3, 59.5, 118.9, 122.1, 122.7, 123.0, 125.8, 126.3, 128.1, 128.6, 128.8, 129.6, 133.2, 134.8, 135.5, 137.2, 138.1, 148.2, 166.5, 168.5, 170.9, 173.4, 173.6; HRMS calcd for C_34_H_39_N_4_O_8_ [M+H]^+^ 646.2877, found 646.2880.

**IId:** white solid, yield 78%. ^1^H NMR (CD_3_OD, 400 MHz) δ: 7.92–7.83 (m, 1H), 7.76 (d, *J* = 7.4, 2H), 7.57 (d, *J* = 5.3, 1H), 7.43 (s, 1H), 7.34 (dd, *J *= 14.3, 7.0, 3H), 7.23 (t, *J *= 7.8, 1H), 7.14 (dd, *J* = 14.2, 7.4, 4H), 7.03 (t, *J* = 7.0, 1H), 4.63 (d, *J* = 9.8, 2H), 4.27 (d, *J* = 7.4, 1H), 3.61 (dq, *J *= 13.2, 6.6, 4H), 3.00 (dd, *J *= 13.7, 8.3, 1H), 2.06 (dd, *J *= 13.6, 6.8, 1H), 1.76–1.52 (m, 3H), 0.95–0.81 (m, 12H). ^13^C NMR (CD_3_OD, 100 MHz) δ: 17.3, 17.9, 18.1, 18.4, 30.5, 37.8, 55.6, 58.9, 59.4, 117.1, 125.8, 127.0, 127.6, 128.1, 128.8, 129.3, 131.3, 134.1, 134.7, 135.7, 137.6, 143.6, 144.3, 161.0, 169.0, 170.5, 171.3, 172.8; HRMS calcd for C_34_H_40_N_4_O_6_ [M+H]^+^ 601.3026, found 601.3029.

**IIe**: white solid, yield 82%.^1^H NMR (CD_3_OD, 400 MHz) δ: 8.06 (d, *J *= 6.2, 1H), 8.03–7.95 (m, 1H), 7.85–7.77 (m, 1H), 7.66–7.59 (m, 1H), 7.56 (d, *J *= 7.8, 1H), 7.49 (t, *J* = 7.8, 1H), 7.44–7.34 (m, 4H), 7.34–7.24 (m, 2H), 7.17 (t, *J *= 7.5, 1H), 4.93 (dd, *J *= 9.3, 4.6, 1H), 4.86 (t, *J* = 7.1, 1H), 4.46 (d, *J *= 7.5, 1H), 4.10 (s, 3H), 3.47 (d, *J* = 4.8, 1H), 3.22 (dd, *J* = 13.8, 9.4, 1H), 2.34–2.24 (m, 1H), 1.89–1.77 (m, 3H), 1.13 (ddd, *J* = 18.5, 8.7, 4.4, 12H); ^13^C NMR (CD_3_OD, 100 MHz) δ: 19.3, 20.7, 22.0, 24.7, 30.8, 36.8, 40.8, 52.3, 55.1, 59.3, 111.5, 118.9, 120.5, 121.1, 122.7, 123.0, 126.3, 128.0, 128.6, 128.9, 130.6, 132.9, 134.9, 137.4, 138.2, 139.5, 157.6, 166.7, 170.7, 171.5, 173.5; HRMS calcd for C_35_H_42_N_4_O_7_ [M+H]^+^ 631.3132, found 631.3136.

**IIf**: white solid, yield 78%. ^1^H NMR (400 MHz, MeOD) δ 7.73 (s, 1H), 7.39–7.41 (d, *J* = 8.6 Hz, 1H), 7.32–7.33 (m, 2H), 7.14–7.22 (m, 3H), 6.98–7.02 (m, 1H), 4.63–4.66 (dd, *J* = 9.5, 4.0 Hz, 1H), 4.22–4.34 (m, 2H), 3.20–3.21 (m, 4H), 2.80–2.83 (m, 1H), 2.47–2.72 (m, 1H), 2.56 (s, 3H), 2.15–2.17 (m, 1H), 1.39–1.76 (m, 8H), 0.71–0.97 (m, 12H); ^13^C NMR (CD_3_OD, 100 MHz) δ:17.2, 18.3, 20.2, 21.9, 23.7, 24.6, 29.2, 30.4, 39.9, 42.5, 51.9, 54.9, 59.2, 67.8, 117.6, 122.5, 122.6, 125.9, 127.7, 128.6, 129.1, 135.3, 138.0, 139.7, 170.0, 170.6, 171.9, 172.1, 173.2; HRMS calcd for C_34_H_46_BrN_5_O_6_ [M+H]^+^ 700.2631, found 700.2636.

**IIg**: white solid, yield 72%. ^1^H NMR (400 MHz, MeOD) δ 7.99 (s, 1H), 7.72–7.74 (d, *J* = 8.6 Hz, 1H), 7.44–7.46 (m, 2H), 7.36–7.38 (m, 2H), 7.15–7.17 (m, 2H), 4.69–4.71 (m, 1H), 4.44–4.47 (m, 1H), 4.35–4.37 (m, 1H),3.73–3.75 (m, 1H), 3.32–3.35 (m, 1H),3.20–3.24 (m, 1H), 2.45–2.52 (m, 1H), 2.44 (s, 3H), 2.16–2.18 (m, 1H), 1.62–1.80 (m, 8H), 1.36–1.37 (m, 2H), 0.91–1.20 (m, 12H); ^13^C NMR (CD_3_OD, 100 MHz) δ: 17.3, 18.4, 20.3, 20.9, 21.8, 23.7, 24.6, 29.3, 30.5, 39.9, 42.5, 51.9, 54.9, 59.3, 65.8, 117.8, 122.5, 122.6, 125.9, 127.7, 128.7, 129.1, 135.3, 138.0, 139.7, 170.0, 170.6, 171.9, 172.1, 173.0; HRMS calcd for C_34_H_46_BrN_5_O_6_ [M+H]^+^ 700.2631, found 700.2634.

## Additional file

**Additional file 1.** The ^1^HNMR and ^13^CNMR spectra of key intermediates and final products were listed.

## Abbreviations

EDC: 1-(3-dimethylaminopropyl)-3-ethylcarbodiimide hydrochloride
TFA: trifluoroacetic acid
DIPEA: *N,N*-diisopropylethylamine
HOBt: 1-hydroxybenzotriazole
DCM: dichloromethane
Boc-l-Val: *N*-Boc-l-Valine
Boc-l-Leu: *N*-Boc-l-Leucine
*l*-Phe-OEt: l-phenylalanine ethyl ester
DMF: *N,N*-dimethylformamide
