# Supplementary material for: Synthesis and antitumor activities of 3-substituted-analine derivatives: structure modifications of Tuv part of tubulysins
Source: Chem Cent J. 2018 Nov 15;12:115. doi: 10.1186/s13065-018-0483-5 (PMC6768038; doi:10.1186/s13065-018-0483-5)

**Synthesis and Antitumor Activities of 3-Substituted -Analine Derivatives: Structure Modifications of Tuv Part of Tubulysins**

Mingsha Shao^a^, Xinfa Bai,^a^ Xuan Ma,^a^ Ning Yan,^a^ Lei Yao *^, a^

^a^ *School of Pharmacy, Key Laboratory of Molecular Pharmacology and Drug Evaluation (Yantai University), Ministry of Education, Collaborative Innovation Center of Advanced Drug Delivery System and Biotech Drugs in Universities of Shandong, Yantai University, Yantai 264005 (P.R. China)*

*^*^ Corresponding author:* [*yaoleiytu@163.com*](mailto:yaoleiytu@163.com)

**Additional Information**

The ^1^HNMR and ^13^CNMR spectra of key intermediates and final products were listed below.


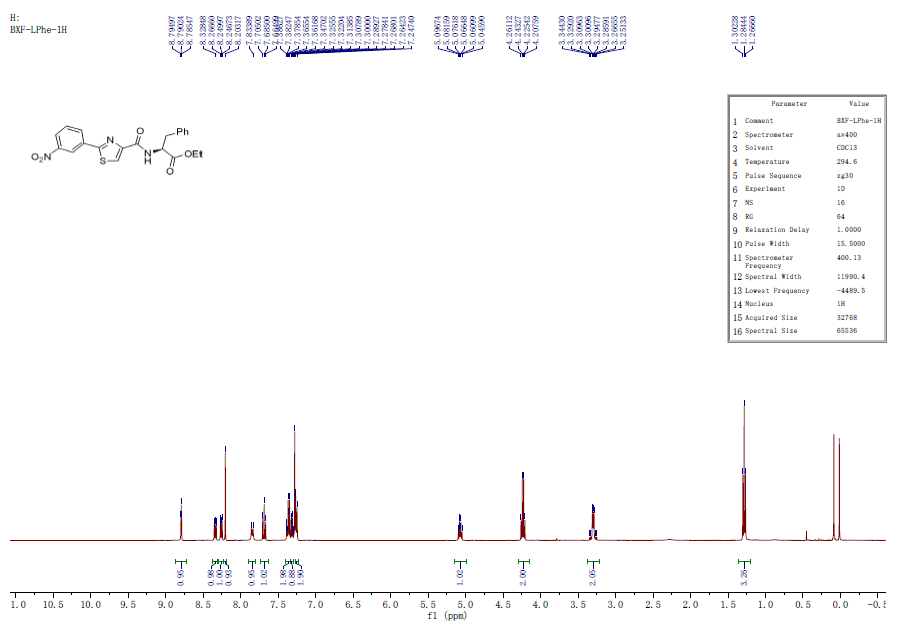


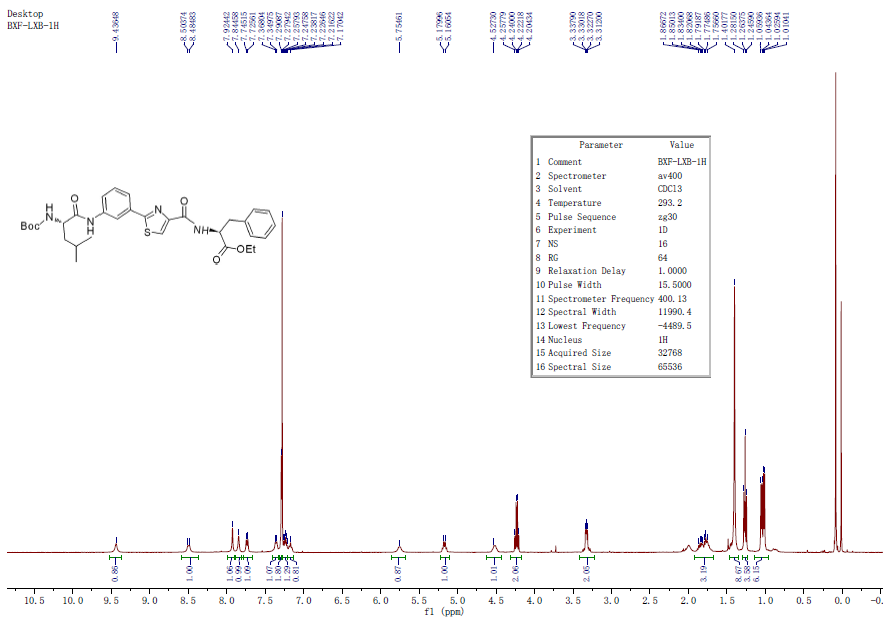


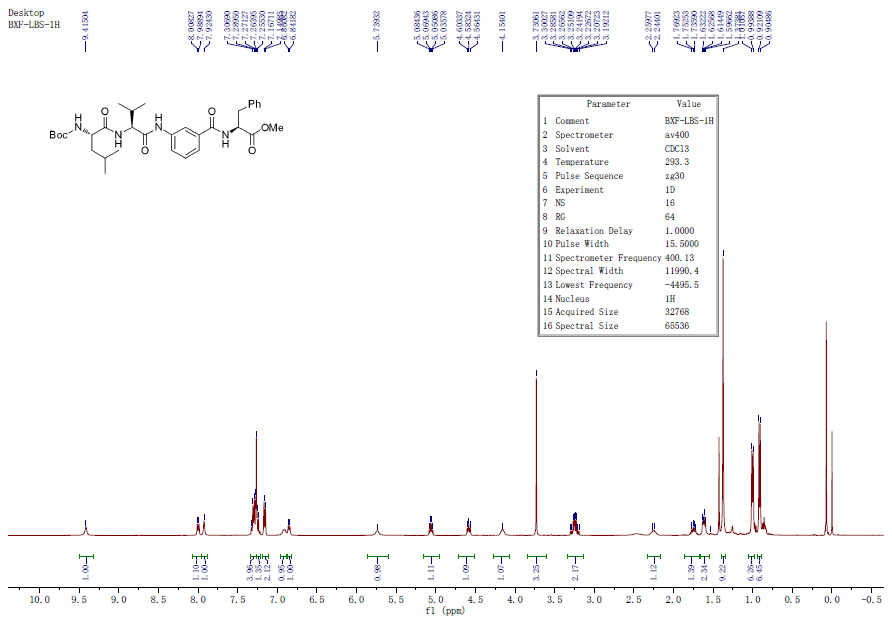


^1^H NMR spectrum of **1a**

^13^C NMR spectrum of **1a**


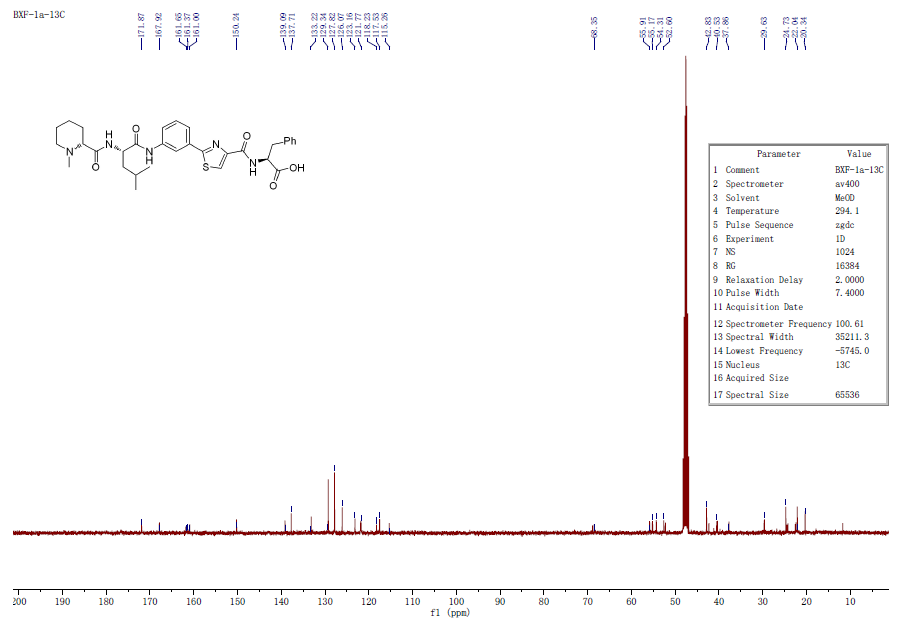


^1^H NMR spectrum of **1b**

^13^C NMR spectrum of **1b**

^1^H NMR spectrum of **1c**

^13^C NMR spectrum of **1c**

^1^H NMR spectrum of 1d


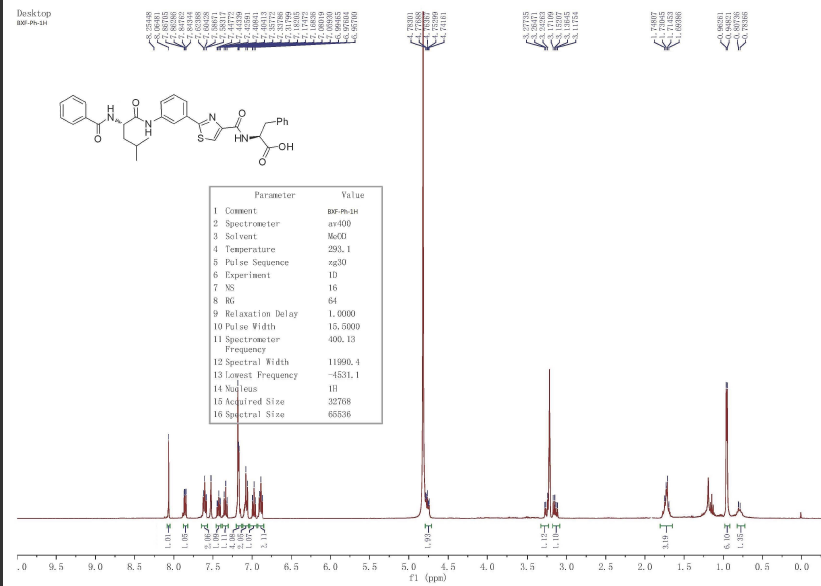


^13^C NMR spectrum of 1d


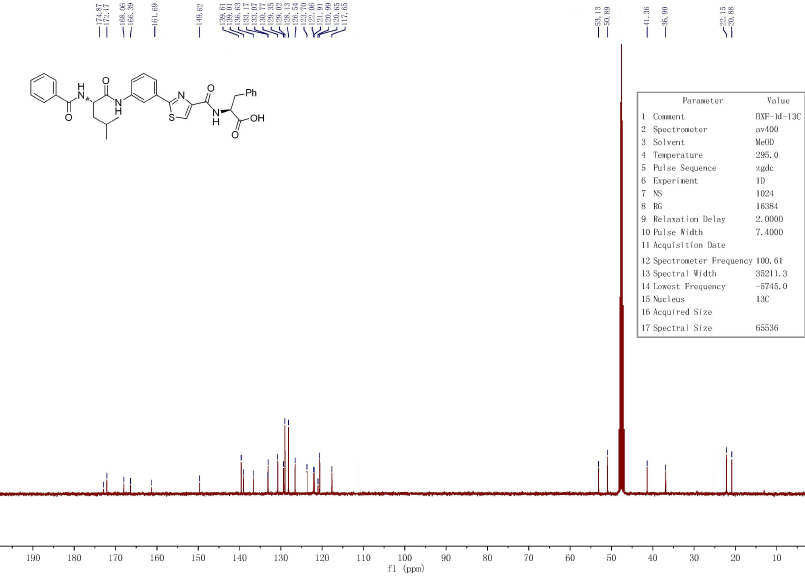


^1^H NMR spectrum of **1e**

^13^C NMR spectrum of **1e**

^1^H NMR spectra of **4**


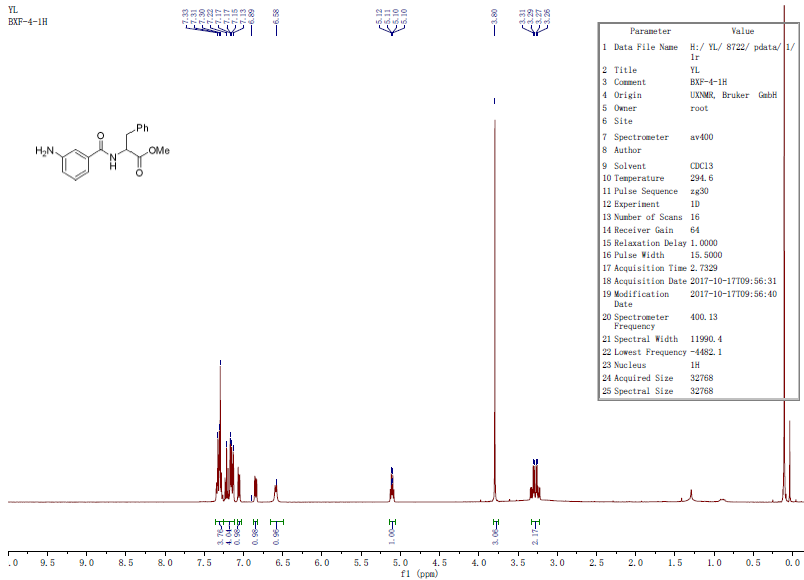


^13^C NMR spectra of **4**


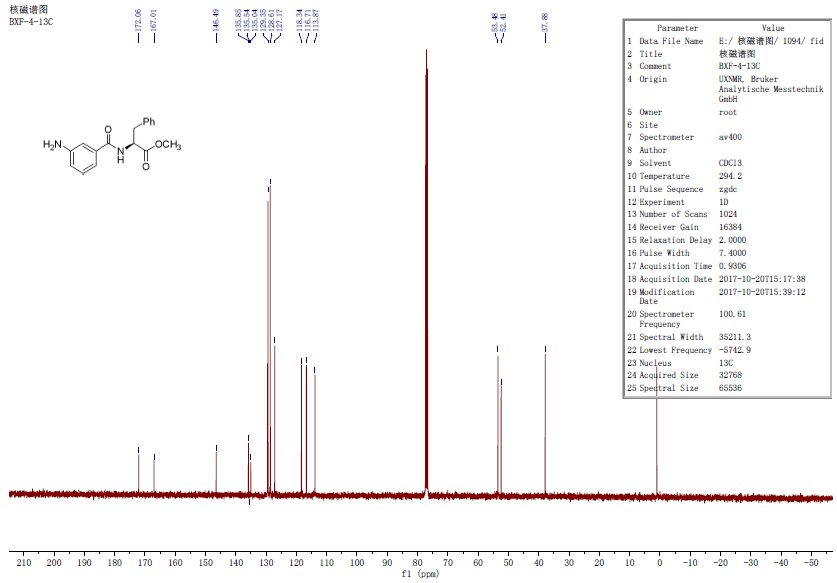


^1^H NMR spectra of **4a**


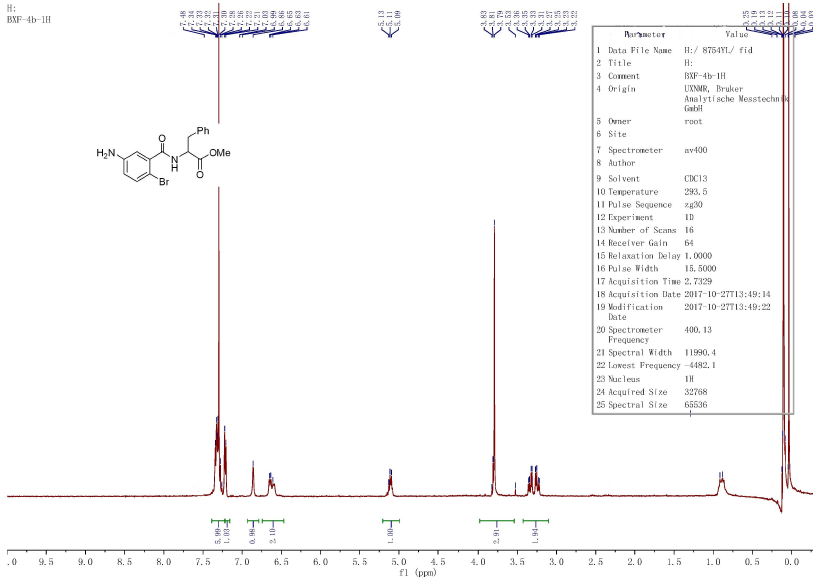


^13^C NMR spectra of **4a**


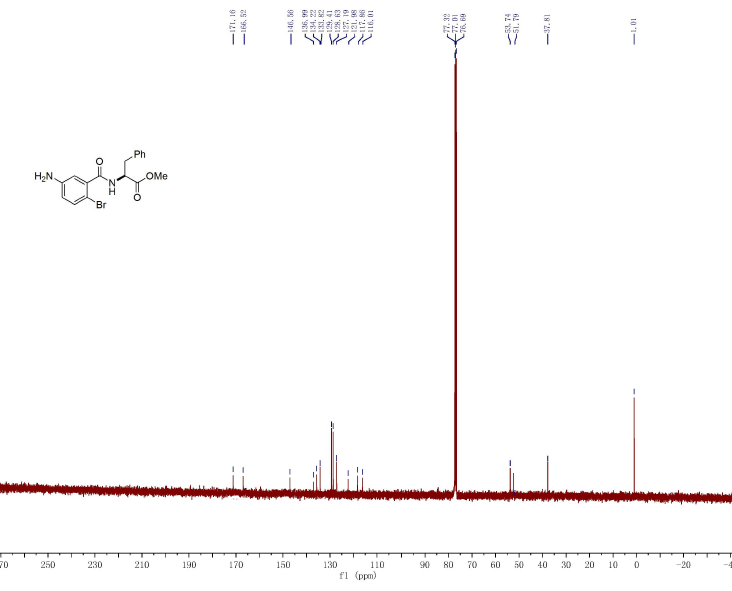


^1^H NMR spectra of **5**


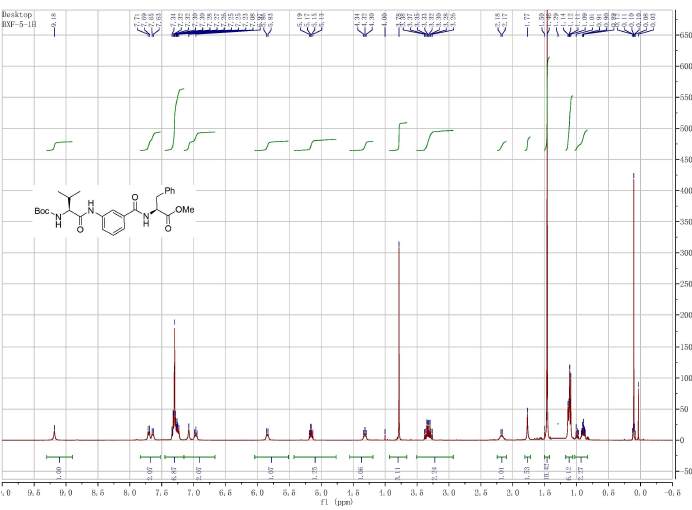


^13^CNMR spectra of **5**


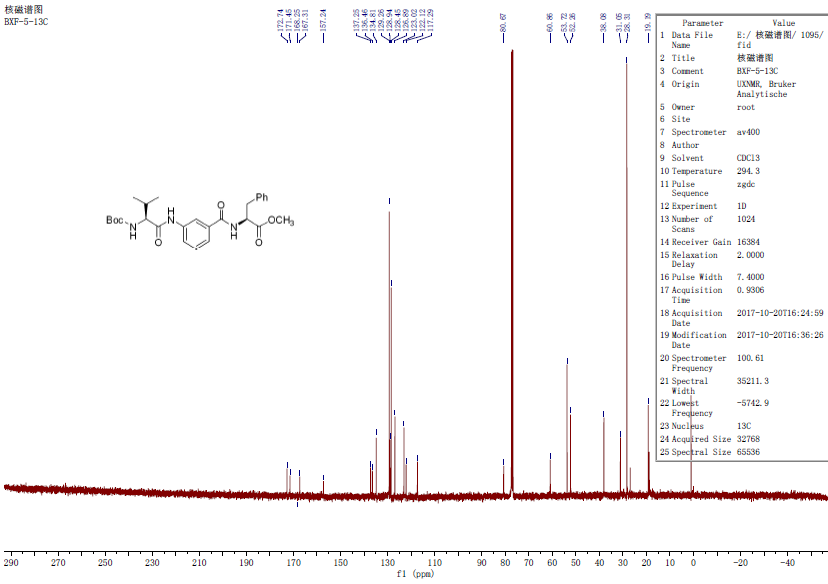


^1^H NMR spectra of **5a**


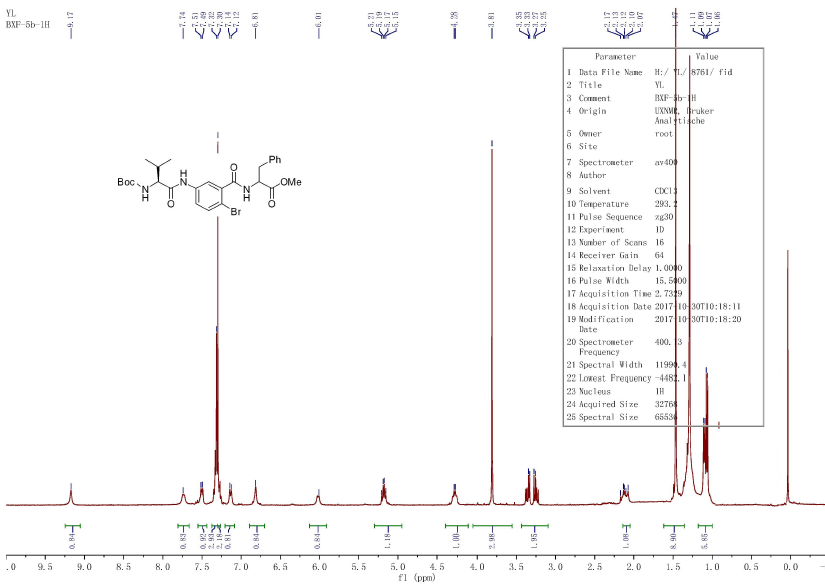


^13^C NMR spectra of **5a**


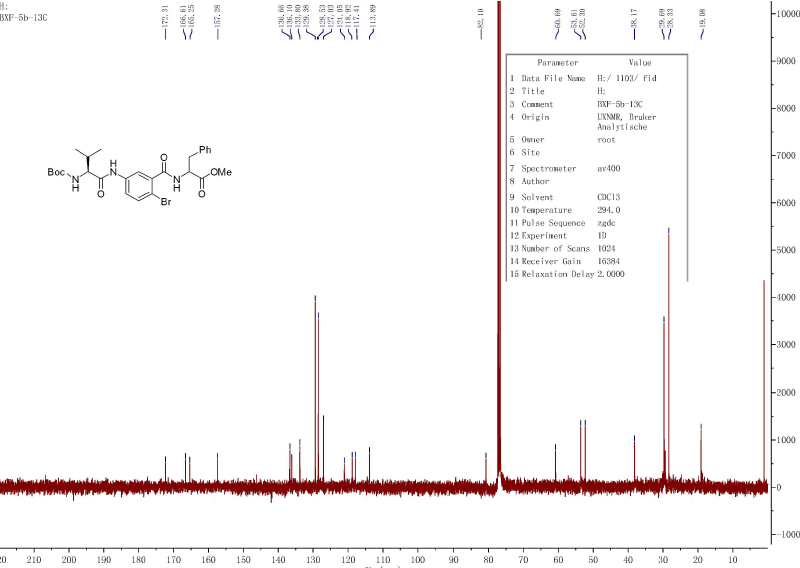


^1^H NMR spectra of **6**


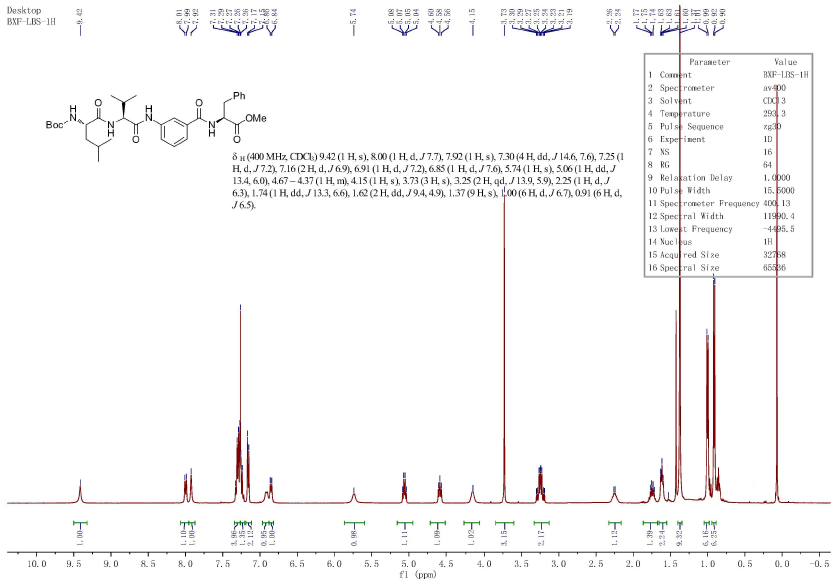


^13^CNMR spectra of **6**


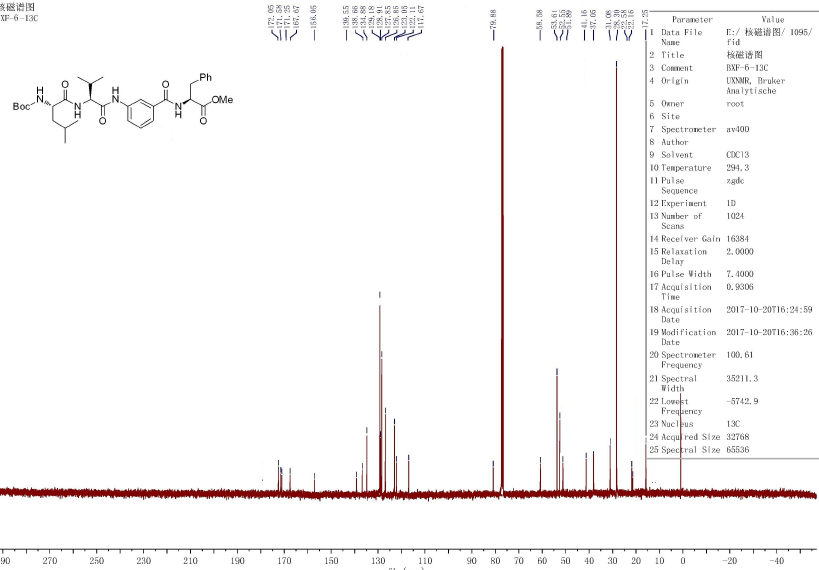


^1^H NMR spectra of **6a**


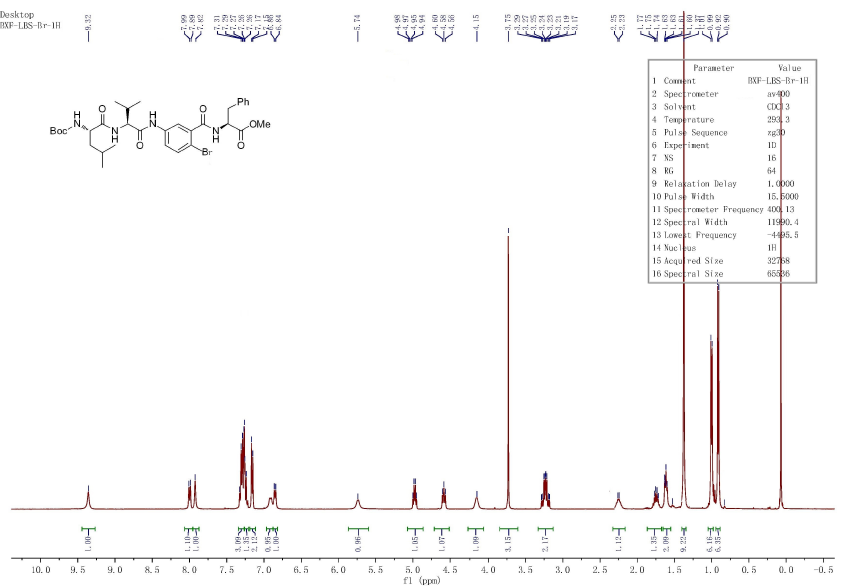


^13^C NMR spectra of **6a**


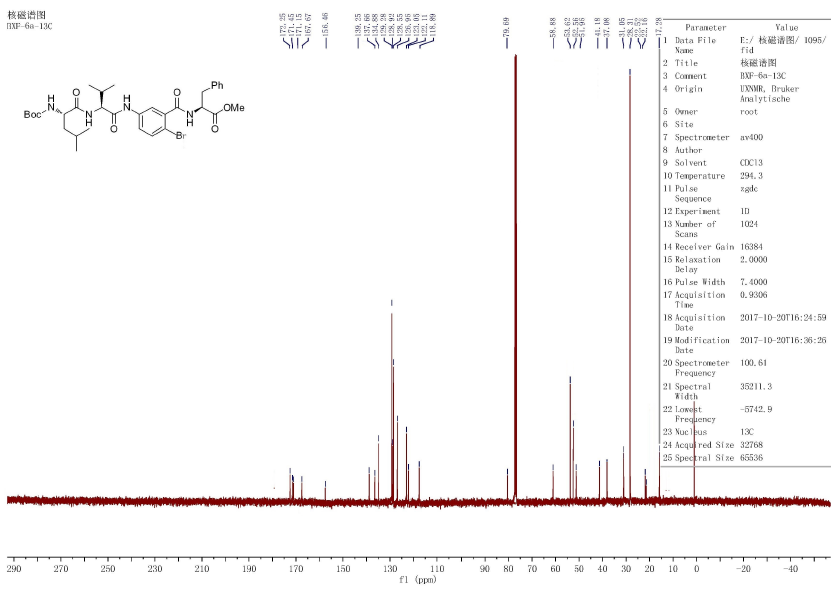


^1^H NMR spectrum of **IIa**

^13^C NMR spectrum of **IIa**

^1^H NMR spectrum of **IIb**

^13^C NMR spectrum of **IIb**

^1^H NMR spectrum of **IIc**

^13^C NMR spectrum of **IIc**

^1^H NMR spectrum of **IId**

^13^C NMR spectrum of **IId**

^1^H NMR spectrum of **IIe**

^13^C NMR spectrum of **IIe**

^1^H NMR spectrum of **IIf**


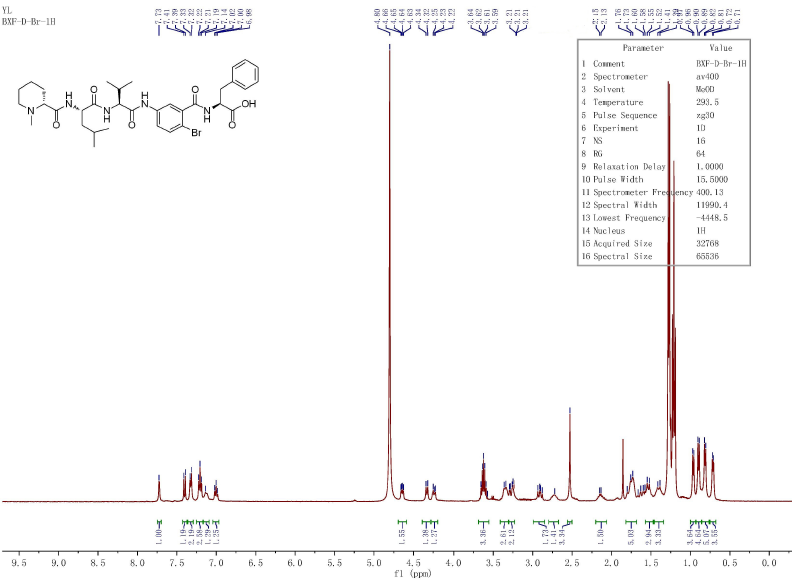


^13^C NMR spectrum of **IIf**


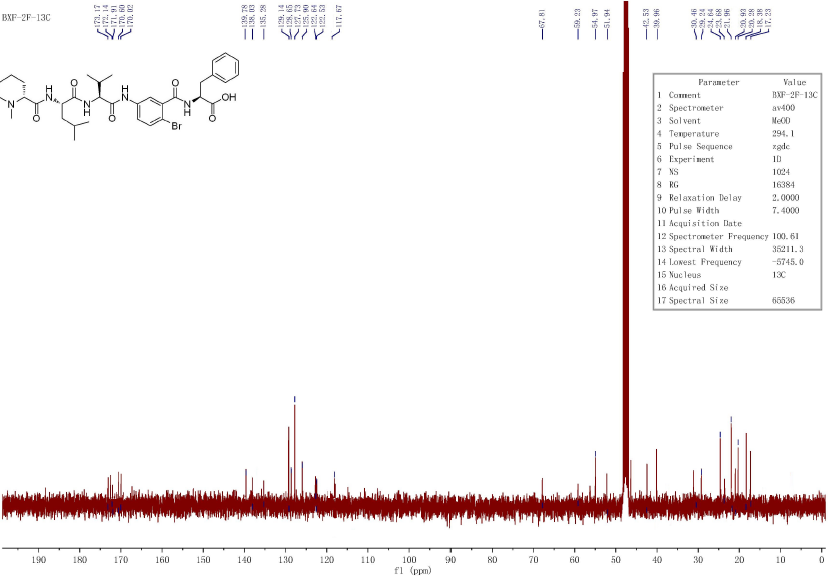


^1^H NMR spectrum of **IIg**


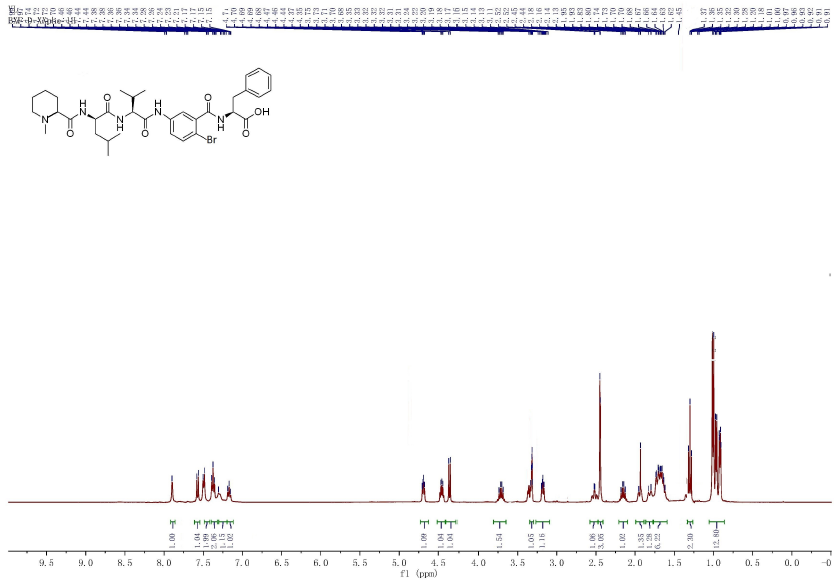


^13^C NMR spectrum of **IIg**


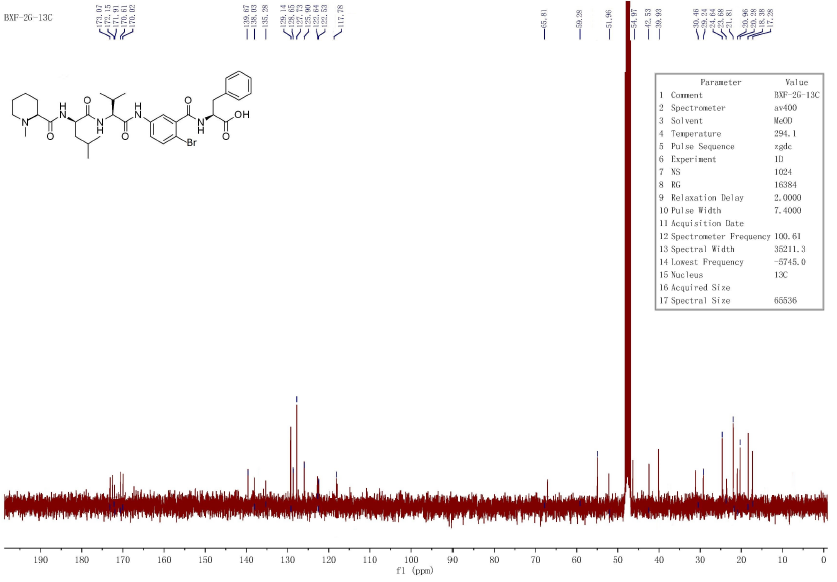

Supplement: Supplementary file 1 — Additional file 1. The 1HNMR and 13CNMR spectra of key intermediates and final products were listed. [file 13065_2018_483_MOESM1_ESM.doc]
